# The complete chloroplast genome and phylogenetic analysis of *Paris stigmatosa* (Melanthiaceae)

**DOI:** 10.1080/23802359.2021.1990146

**Published:** 2021-10-14

**Authors:** Shu-Dong Zhang, Xiu-Fei Yang, Qin Wang, Mao-Mao Du, Li-Zhen Ling

**Affiliations:** School of Biological Science and Technology, Liupanshui Normal University, Liupanshui, China

**Keywords:** *Paris stigmatosa*, Melanthiaceae, chloroplast genome, phylogenetic analysis

## Abstract

*Paris stigmatosa* is a new described species of Melanthiaceae. In this study, the complete chloroplast (cp) genome sequence of *P. stigmatosa* was first reported and characterized. The cp genome is 165,623 bp in length and contains a pair of inverted repeats (IRs, 34,165 bp) separated by a large (84,327 bp) and small (12,966 bp) single-copy regions. A total of 113 genes were predicted, including 79 protein-coding genes, 30 tRNA genes and 4 rRNA genes. The phylogenetic analysis suggested that *P. stigmatosa* is sister of the clade formed by *P. marmorata* and *P. luquanensis*.

The genus *Paris* (Melanthiaceae) comprises 27 species accepted in The Plant List (2013) and divides into two subgenera *Paris s. s.* and *Daiswa* (Huang et al. 2016). *Paris* is well known in China for its medicinal qualities. In recent years, some new *Paris* species were gradually reported, such as *P*. *lihengiana* (Xu et al. 2019), *P. tengchongensis* (Ji et al. 2017) and *P*. *nitida* (Wang et al. 2017). *Paris stigmatosa* Shu D. Zhang is a new species described in 2008 (Zhang et al. 2008). This species is very similar to *P*. *polyphylla* in the morphology, but it has longer stigmas (21-34 mm). *Paris stigmatosa* is only geographically found in Yaoshan Mountain of Yunnan Province, China (Zhang et al. 2008). Until now, little information is known about this species. To better understand and utilize this species, we sequenced and analyzed the complete chloroplast (cp) genome of *P. stigmatosa* using high-throughput sequencing technology.

The specimen (03-1681) was collected from Yaoshan Mountain (Qiaojia, Yunnan, China; 103°00′E, 26.52′N) and deposited at Herbarium, Kunming Institute of Botany, CAS (KUN, http://www.kun.ac.cn/, Tao Deng, dengtao@mail.kib.ac.cn). Genomic DNA was extracted with a modified CTAB (Cetyl Trimethyl Ammonium Bromide) method (Yang et al. 2014) from the fresh leaves. Purified DNA was fragmented and used to construct short-insert (350 bp) library using NEB Next Ultra DNA Library Prep Kit for Illumina (NEB, USA) as per manufacturer’s recommendations. Approximately 6 Gb raw data of 150 bp paired-end reads were generated using the Illumina HiSeq X ten platform at Beijing Novogene Bioinformatics Technology Co., Ltd. (Nanjing, China) and used for the cp genome assembly using SPAdes (Bankevich et al. 2012). The cp genome annotation was accomplished using PGA (Qu et al. 2019) with the cp genomes of *P. marmorata* (KX784047) and *P. thibetica* (KY247143) as reference sequences coupled with manual check and adjustment. The circle cp map of *P. stigmatosa* was generated by OGDRAW (Greiner et al. 2019).

The complete cp genome of *P. stigmatosa* (accession number MN723866) is 165,623 bp in length with a typical quadripartite structure containing two inverted repeats (IRs) of 34,165 bp, a large single copy (LSC) region of 84,327 bp and a small single copy (SSC) region of 12,966 bp. The overall GC content of the cp genome is 36.8%. A total of 113 unique genes consist of 79 protein-coding genes, 30 transfer RNA (tRNA) genes, and 4 ribosomal RNA (rRNA) genes, which is little different from other species of *Paris* (Huang et al. 2016). Among these genes, 15 genes (*atpF*, *ndhA*, *ndhB*, *petB*, *petD*, *rpl16*, *rpl2*, *rpoC1*, *rps16*, *trnA-UGC*, *trnG-UCC*, *trnI-GAU*, *trnK-UUU*, *trnL-UAA*, *trnV-UAC*) contain one intron and three genes (*clpP*, *rps12* and *ycf3*) have two introns.

In this study, we constructed the phylogenetic tree and analyzed the phylogenetic position of *P. stigmatosa* based on the maximum likelihood (ML) and Bayesian inference (BI) methods (Ronquist et al. 2012; Stamatakis 2014). Five species from *Trillium* (*T. tschonoskii*, *T. decumbens*, *T. maculatum*, *T. cuneatum* and *T. govanianum*) were used as the outgroups. The cp genomes of *P. stigmatosa* and previously published species of *Paris* were used for phylogenetic analysis. The complete cp genome sequences were aligned by using MAFFT version 7.308 (Katoh and Standley 2013). The best-fit model (TIMef) for the dataset was determined by MODELTEST v.3.7 (Posada and Crandall 1998) with the Akaike Information Criterion (AIC) (Posada and Buckley 2004). BI was performed with Mrbayes v.3.2 (Ronquist et al. 2012). Two independent Markov Chain Monte Carlo (MCMC) chains were run, each with three heated and one cold chain. Each chain started with a random tree, default priors and sampling trees every 100 generations, with the first 25% discarded as burn-in. Stationarity was considered to be reached when the average standard deviation of split frequencies was <0.01. The ML analysis was performed with RAxML v.8.2.4 (Stamatakis 2014). The ML tree was inferred with the combined rapid bootstrap (1,000 replicates) and search for ML tree (the ‘-f a’ option). The GTRGAMMA model was used in the analysis as suggested (RAxML manual). The phylogenetic analysis showed that *Paris* is monophyletic and can be divided into two separate clades (Figure 1), corresponding to the subgenera *Paris s. s.* and *Daiswa* (Huang et al. 2016). In *Daiswa* clade, *P. marmorata* and *P. luquanensis* are united in one clade and *P. stigmatosa* is positioned as a sister group to this clade (Figure 1).

**Figure 1.**
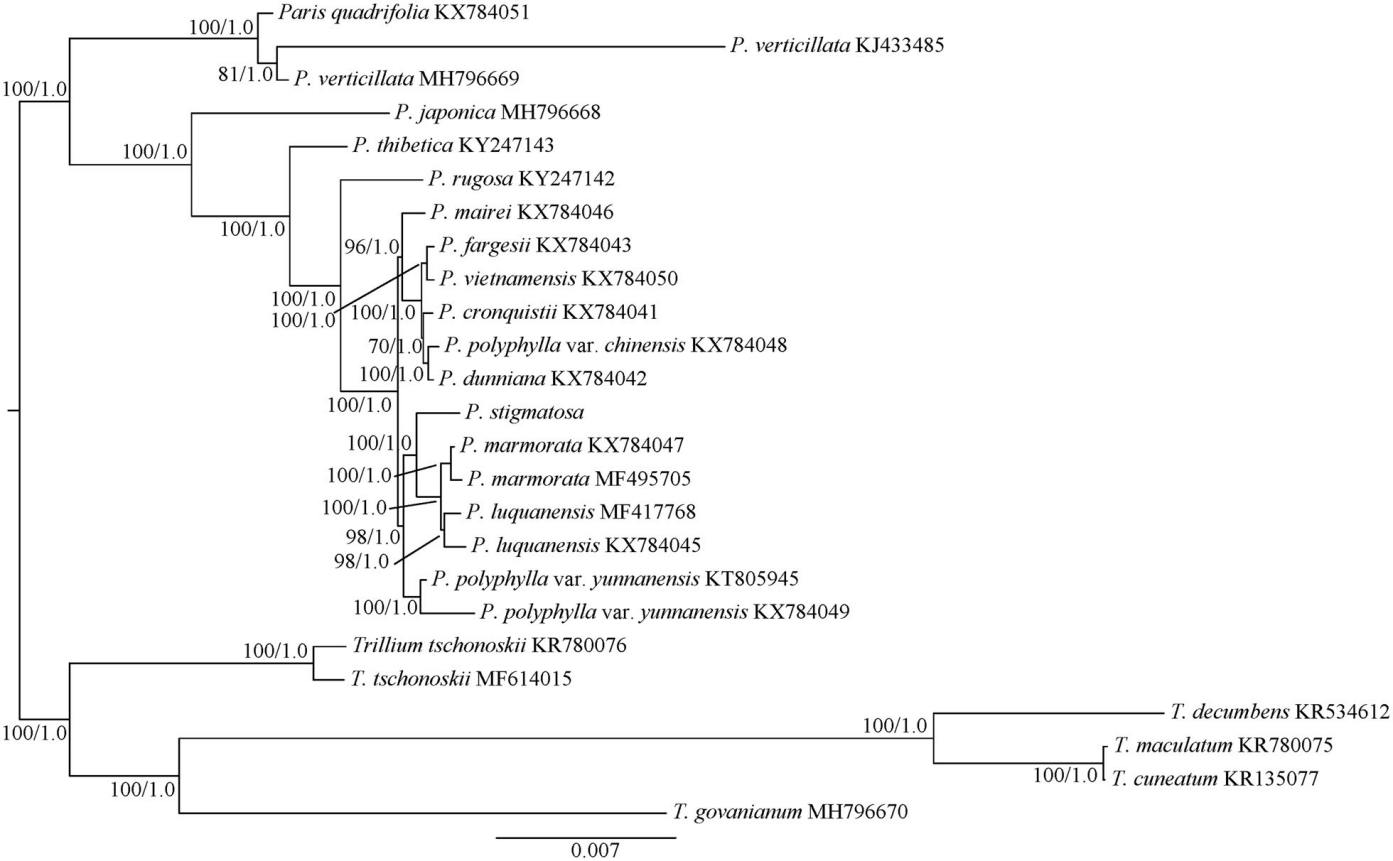
The maximum likelihood (ML) tree of 15 species from *Paris* inferred from the complete chloroplast genome sequences. Numbers at nodes correspond to ML bootstrap percentages (1,000 replicates) and Bayesian inference (BI) posterior probabilities.

## Data Availability

The genome sequence data that support the findings of this study are openly available in GenBank of NCBI at https://www.ncbi.nlm.nih.gov (https://www.ncbi.nlm.nih.gov/) under the accession no. MN723866. The associated BioProject, SRA, and Bio-Sample numbers are PRJNA724898, SRR14420049, and SAMN19009008 respectively.
